# Natural cold freshwater temperatures and light regimes in Atlantic salmon smolt production can improve the sea phase performance

**DOI:** 10.1016/j.vas.2026.100579

**Published:** 2026-01-26

**Authors:** Øystein Klakegg, Henning Sørum

**Affiliations:** a Faculty of logistics, Molde University College, Campus Kristiansund, Fosnagata 12, 6509 Kristiansund, Norway; b Faculty of Veterinary Medicine, Department of Paraclinical Sciences, Norwegian University of Life Sciences, Elizabeth Stephansens vei 15, 1433 Ås, Norway

**Keywords:** Light, Temperature, Health, Sustainability

## Abstract

A total of 5.4 million smolts of Atlantic salmon (*Salmo salar*) older than 15 months, were divided into 18 groups and followed after sea-launch. They were all produced in the freshwater stage by employing natural conditions including water temperature and light program. The smolt were reared at water temperatures that in winter is as low as +3 °C and is referred to as “cold smolt”. The 18 groups were transferred to 11 net pen locations in Norway. Mortality, Feed Conversion Ratio (FCR) and share superior of the 18 groups were analyzed after harvesting and compared with the average in the respective production regions (POs) where the fish had been located. When the “cold smolt” had been co-located with the conventionally produced smolt groups, the “cold smolt” where also compared with these.

The results indicate that the "cold smolt" performed better in the sea phase than both the average of all smolt in the respective production areas and compared to other smolts released at the same time at the same locations. Compared to the annual average in the same PO the “cold smolt” performed, from 2 % to 74 % better regarding mortality, from 4 % to 22 % better regarding FCR and from 2 % worse to 49 % better regarding superior product quality.

## 1 Introduction

The culture of Atlantic salmon in Norway, both in the freshwater phase and in the seawater phase, differs considerably from the natural conditions for wild Atlantic salmon, particularly in winter in the freshwater stage with the use of higher water temperature and more use of extended or continuous light exposure.

Wild Atlantic salmon in Norway spawn naturally in the rivers from October to December, and the eggs lie buried in the river gravel until they hatch in April/May. The yolk sac is consumed in the spring before the fry starts to consume external feed from the river in the summer (Thorstad et. al., 2010).

After one to eight years in the Norwegian rivers the salmon smoltify and migrate to the sea. The fish is then between 12 to 18 cm long and 10-80 g in weight (Harvey et al., 2020; Thorstad et. al., 2010).

There is a tendency for wild salmon to smoltify at an older age further north, but also in southern Norway, salmon can stay six years or more in the rivers before they migrate into the sea (Heggberget et al., 1986; Skaala et al., 2012).

Since the start of aquaculture with Atlantic salmon, light and temperatures have been used to manipulate the salmon, especially in the freshwater phase. This is to obtain faster growth and continuous production all year round (Migaud et al., 2013).

There has been a constant commercial development to reduce production time from hatching to smoltification in the commercial production of Atlantic salmon, which has resulted in production of 0-generation salmon smolt i.e. smolt released into sea the same year as it is hatched, or, after the breeding salmon is manipulated to spawn all year around, less than 10 months after hatching (Stefansson et al., 2005; Bergheim A. et al., 2009). In average during 2010-2012 it was released 55 % 1-year old smolt and 45 % 0-year smolt (Nystøyl et al., 2013).

The 2018 generation of Atlantic salmon in Norway was the first generation of farmed Atlantic salmon of which the largest portion of the population was sea-launched without a winter after hatching (0-generation) (personal communication, Ragnar Nystøyl, Kontali). Atlantic salmon is, like most other fish, poikilothermic and temperature increase lead most often to faster growth (Guderley H. 2004; Lindmark et al., 2022). Therefore in the effort to steadily reduce the production time of Atlantic salmon from spawning to slaughter, the temperature has been increased (Migaud et al., 2013). This is especially true in the roe and hatchery phase, where there is feasible to control temperature. The difference between 12 and 14 °C is 3-4 weeks from start-feeding up to the size of 20 g (Stefansson et al., 2005). For salmon egg, the incubation temperature should be below 8 °C to avoid problems as deformities (Ørnsrud et al., 2004; Ytteborg et al., 2010; Fraser et al., 2015). However, 8 °C is well above the natural temperature in most salmon rivers during the egg period. It is common for the salmon eggs to lie in the river gravel in Norwegian rivers while there is a layer of ice on the river, and the temperature is often 3 °C in the rivers in southern Norway and colder further north (Jonsson and Jonsson, 2014). Based on the experience of many fry certificates, the usual incubation temperature of farmed salmon eggs in Norway is between 6,5 og 7,5 °C. Smoltification in salmon is initiated by increasing day-length (Stefansson et al., 2012; McCormick, 2013; Handeland et al., 2014). The brain registers changes in day length through a light-brain-pituitary axis that is activated with increasing day length (Ebbesson et al., 2003) which eventually leads to the morphological, behavioral and physiological changes in smoltification (Björnsson et al., 2011; McCormick, 2013).

Fish, including Atlantic salmon, have in addition to the circadian clock, a circannular clock, and temperature and lighting manipulate spawning and smoltification (Doyle and Menaker, 2007; Migaud et al., 2013; Sua-Cespedes et al., 2021; Eilertsen M. et.al., 2024). Light seems to be particularly important for the regulation of the circadian clock partly via regulation of the melatonin production in the pineal gland (Doyle and Menaker 2007).

It is not evident what controls the circannular clockwork, but a theory is that epigenetic control of chromatin structures which, with the help of changes in DNA and histone proteins that manifest themselves in active summer genes or silent winter genes, is part of the explanation (Lincoln, 2019; Stevenson and Lincoln, 2017).

In recent years, there have been indications that smolt farmed in cold water and with a more natural light exposure over a longer period of 1.5 -2 years after hatching have performed better than smolt farmed in warm intake-water for less than one year and often with continuous light (Frisk et al., 2020). In recent years, the 1-year smolt in general have also performed better in the sea-phase in terms of production yield than the 0-year smolt. The smolt yield (kg harvest per smolt sea-launched) for 2022 was 4,1 versus for 1-year smolt versus 3.7 kg for 0-year smolt, and for 2023 4,0 versus 3,5 kg. (Personal communication Ragnar Nystøyl, seminar Kristiansund, 17^th^ January 2025, Kontali).

In a study in southern Norway it was observed that post-smolt of Atlantic salmon cultured in a RAS facility with brackish water (14 ‰) in 12 °C had a greater weight gain in the sea phase due to the gain of muscle tissue compared to fish cultured in brackish water with 14 °C (Flo et al., 2025). In another study in Tasmania it was demonstrated that Atlantic salmon farmed at an average temperature of 6 °C in a flow-through facility revealed higher growth after transfer to sea than fish farmed at 14 °C in a RAS facility (van Rijn et al., 2020).

In a study growth performance and environmental adaptation between Atlantic salmon reared in Recirculating Aquaculture System (RAS) and in flow through system were compared. It was found that the RAS fish had lower growth and lower response to stressful environmental variability and increased mortality after sea-launch to slaughtering (Lai et al., 2024). It this trial the water temperature in the flow through group was lower than in the RAS group the last five months before transfer to sea, down to 3,1 °C in the flow through system compared to down to 9,0 °C in the RAS system (Lai et al., 2024). Their hypothesis was that salmon in RAS, which was exposed to a very stable environment, may have an impact on the physiological plasticity which could make the fish less able to cope with the major changes found in the cages throughout the year (Lai et al., 2024).

Biorhythms seem to play an important role in fish, and light and temperature are considered the most important movers of biorhythms (Doyle and Menaker, 2007; Migaud et al., 2013; Sua-Cespedes et al., 2021; Eilertsen M. et.al., 2024). Over millions of years, organisms have adapted to the external living conditions such as light and temperature. By rapidly changing light and temperature conditions, as in intensive smolt farming, the biorhythms are also changed. Perhaps, in our quest for fast-growing smolts, the industry has gone too far regarding increasing temperature and light conditions and removing the salmon too far away from their natural growth conditions with physiologically negative consequences as a result.

In this field-study we focus on farming conditions in freshwater and what significance they have for the Atlantic salmon performance in the sea phase.

Our hypothesis was that smolts produced in conditions that are closer to the natural conditions, more slow-growing than usual, due to low water temperature during winter and with more natural light than most smolt producers use, would perform better in terms of survival, slaughter quality and feed consumption in the sea phase.

## 2 Materials and methods

### 2.1 Description of data collection

The commercial Atlantic salmon smolt groups included in this study were all produced with the use of more than 15 months from hatching to sea launch.

Until September 2020 the whole production from eye eggs to smolt was conducted at one site (Facility1). Smolt that were sea-launched from April 2021 and on had the freshwater period split between two sites with one exception. The smolt group sea-launched in PO 2 in September 2021 were kept the whole freshwater period at Facility 1 (Table 2).

The two sites are located in the same municipality. They have different water sources, but use similar production protocols, including low water temperatures in winter. The production of smolt that were sea-launched from April 2021 were typically housed 10-15 months growing up to the size of 60 – 80 g at Facility 1 before they were transferred to Facility 2 where the smolt grew further (120-300 g, Table 2) until vaccination and sea-launch.

The smolt sea-launched in the autumn had typically been 4-5 months at Facility 2 while the smolt that were sea-launched in the spring/summer typically were kept 8-10 months at Facility 2. The main difference between Facility 1 and 2 is that Facility 2 had colder water temperature during the summer (Table 1).

**Table 1:** Water temperature at Facility 1 and Facility 2.

	Average water temperature ^o^C		
	Facility 1		Facility 2
	2021 and earlier	2022*	From 2021
Jan	3	3	3
Feb	3	3	3
March	3	3	3
April	5	5	5
May	8	8	9
June	14	13	11
July	15	14	12
Aug	17	14	12
Sept	16	14	12
Oct	11	11	10
Nov	6	6	8
Dec	3	3	4

The winter temperature (3 °C) of the rearing water in the freshwater Facility 1 and 2 is clearly lower than the average temperature in freshwater aquaculture facilities in Norway which is often 12-14 °C (Stefansson et al., 2005, Stefansson et al., 2012).

The reason for the colder summer water temperatures after 2021 is that Facility 1 made a deeper water intake in the spring of 2022.

The light exposure at Facility 1 was continuous light during initial feeding of the fish group with the start of the initial feeding in June. The fish group with the start of the initial feeding in August had only a few hours without light and a few additional hours without light for the fish group with the initial feeding starting in October. After the initial feeding the light regime was 12h/12h light/dark.

At Facility 2 the light exposure was 10h/14h light/dark after they receive the salmon, and 24h light before delivery to the sea sites.

Both facilities use a flow-through freshwater supply without heat exchange. None of the facilities disinfect the raw water. Facility 1 has flow-through of all the water, Facility 2 uses 1/3 new raw water and 2/3 reused water, which has passed CO^2^ degasser.

**Fig. 1:**
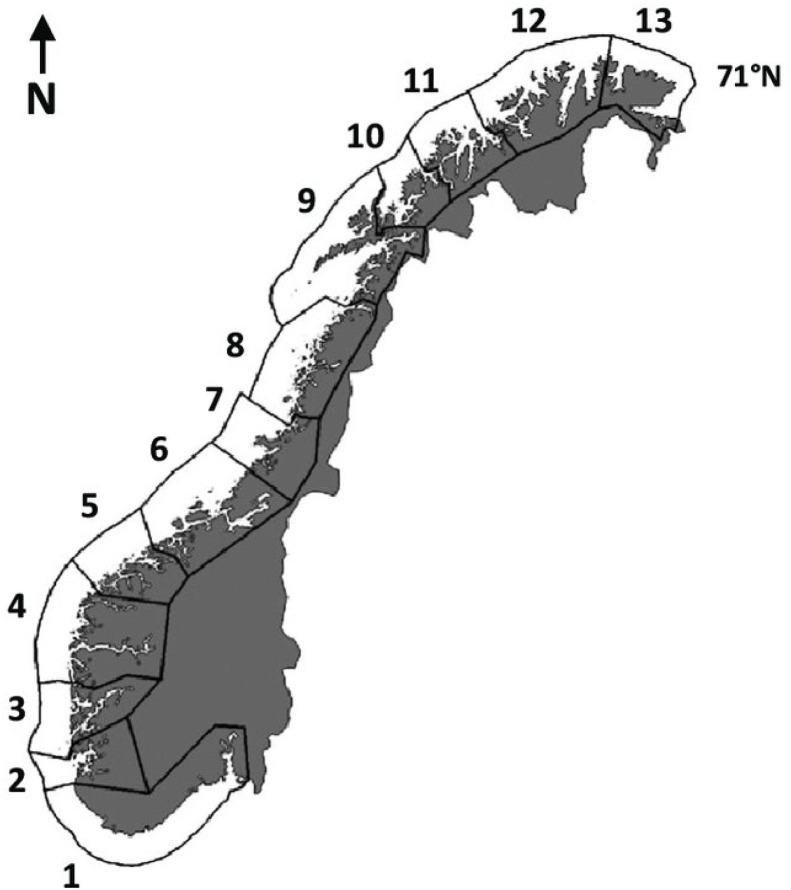
Atlantic salmon aquaculture production areas (1-13) in Norway. Average figures for FCR, mortality and share superior are prepared by Norwegian Directorate of Fisheries and Norwegian veterinary Institute for each production area. The figures vary between production areas, it is therefore relevant to compare the results in relation to production area and not in relation to the whole country. Ministry of Trade, Industry and Fisheries, 2017). License: https://creativecommons.org/licenses/by/4.0/legalcode.en.

Data were collected from 18 groups of smolt from 11 grow-out sites in seawater belonging to five different fish farming companies from Sogn og Fjordane, Hordaland and Rogaland using “cold smolt” (Table 2). The data were developed in the Production areas (PO) 2, 3 and 4. In Norway the coast is divided in a total of 13 production areas, see Fig. 1.

**Table 2:** The economic feed conversion ratio (FCRe), mortality and share “superior” in 18 groups of “cold” smolt launched from October 2018 to September 2022 at 11 production sites in 3 production areas.

Month of hatching	Freshwater period	Month of sea-launch	Smolt weight (g)	Number of smolt (1000)	Mortality at sea(%)	FCRe	Superior (%)	Production area	Company	Production site
Jul 2017	Facility 1	Oct 2018	65	227	3,4	1,13	82	2	1	1
Jul 2017	Facility 1	Aug 2019	97	356	7	1,11	79	4	4	8
Jun 2018	Facility 1	Sept 2019	99	226	13,4	0,99*	90	2	1	2
Jul 2018	Facility 1	April 2020	150	527	28,6	1,43	93	4	3	6
April 2018	Facility 1	Aug 2020	186	282	10	1,12	90	4	4	7
April-May 2018	Facility 1	Sept 2020	178	442	7	1,22	95	3	2	3
April 2019	Facility 1 and 2	April 2021	278	461	11,6	1,10	87	3	2	4
Jun 2019	Facility 1 and 2	April 2021	189	441	11,9	1,19	90	4	4	8
Sept 2019	Facility 1 and 2	Jul2021	116	262	19,9	1,23	93	2	1	2
Sept 2019	Facility 1 and 2	Sept 2021	306	143	5	1,08	92	4	4	9
April 2020	Facility 1 and 2	Sept 2021	158	243	3,7	1,07	92	4	4	9
May 2020	Facility 1	Sept 2021	200	248	3,3	1,19	96	2	1	2
May 2020	Facility 1 and 2	Sept 2021	251	399	7	1,22	90	3	2	5
June 2020	Facility 1 and 2	May 2022	117	390	8,7	1,13	70	4	4	10
Sept 2020	Facility 1 and 2	Aug 2022	200	89	9,1	1,07	90	4	4	7
April 2021	Facility 1 and 2	Aug 2022	179	238	10,1	1,21	88	4	4	7
April 2021	Facility 1 and 2	Sept 2022	171	184	11,8	1,13	91	4	5	11
April 2021	Facility 1 and 2	Sept 2022	145	229	8,3	1,28’'	95	2	1	1

*This group of fish had an incidence of high mortality after sea launch and where also harvested around 2.5 kg., due to a public requirement caused by a contagious disease at a nearby location.

**The high FCRe in the fish group in Company 1 sea launched September 2022 is due to the high average harvest weight which was 9.0 kg.

We followed 5.4 million “cold smolt” from sea launch to slaughter.

Three of the farmers had only “cold smolt” at the same location simultaneously, two of the farmers had both “cold smolt” and other faster grown (0-year and 1-year old) smolt at the same location during the same period; in total five groups.

The 0-year faster grown smolt were raised in a facility using a flow-through freshwater supply with heat exchange. The 0-year smolt were sea-launched 9-11 months after hatching. The temperature during initial feeding was 13-14 °C, and 13-18 °C from initial feeding to transfer to sea. The light regime for the 0-year smolt was 24h light until 3 months before transferring to sea, then about 8 weeks with 12h/12h light dark and 4 weeks 24h light before sea launch. Some of the 0-year smolt were bathed in probiotic bacteria (Stembiont^TM^) at the same time as vaccination. The eggs came from AquaGene and SalmoBreed. All together we followed 1,43 million faster grown smolt from sea launch to slaughter (Table 3).

**Table 3:** Locations that had both “cold smolt” and other smolt (red digits) at the same site simultaneously (all these locations were at Production area 4).

* This fish were 1-year old at sea-launch.

The data were compared between the fish groups at the same location. The production data were in addition compared between fish groups in the same period within the respective production area.

In the Norwegian standard for fish (NBS 10-01) the Superior class of slaughtered Atlantic salmon (“superior”) is a product without substantial faults, damages or defects with skin that is shiny and with no significant loss of scales, no healed sores impairing the overall impression, no open sores, and with whole or worn (healed) fins.

Yearly quality categorization of the slaughtered farmed salmon for the various production regions in Norway was not available until 2023, therefore we contacted the Norwegian Food Safety Authority and received all slaughter reports from all 16 salmon slaughterhouses operating from the end of 2016 to December 2023 from Møre og Romsdal and southward (PO 2, 3, 4 and 5). We estimated that the fish were farmed in the same farming area as the slaughterhouse was located. Of the more than 10.000 slaughter plant data (slaughter days) we got, we deleted 5 days that seemed unlikely (more than 10 times normal daily volume slaughtered). The quality categorization of the slaughtered farmed salmon in the different regions from 2019-2022 based on the harvest data from Norwegian Food Safety Authority is shown in Table 3. The data from 2023 is taken from the annual Fish Health Report (Fiskehelserapporten), which in turn has received the data from the Norwegian Food Safety Authority (Sommerset et al., 2024). The economic feed conversion ratio (the efficiency of converting feed into marketable fish, dividing the total amount of feed used by the total harvested weight of marketable fish) was selected from The Fishery Authority (Norwegian Directorate of Fisheries, 2024). These data do not fit fully with the producer area but are county-wise. There have also been changes in the county structure during the period as Sogn og Fjordane and Hordaland have been merged into Vestland county. In 2023 The Fishery Authority only reported numbers for whole Norway, and not for each county.

The mortality figures for the producer areas, as a percentage, are taken from the Norwegian Veterinary Institute, and Fish Health Report for each year (Sommerset et al. 2021; Sommerset et al. 2022; Sommerset et al. 2023; Sommerset et al. 2024).

For the out-growing farmers, farmers that have the fish in the saltwater phase from smolt to harvest/slaughtering, we have used data after the whole group of fish have been harvested – then we have the total figures for both feed factor (economical), mortality and proportion of superior. Mortality was calculated by taking the total number of dead from sea-launch to slaughter, divide this by the total number of fish sea-launched and then multiply by 100 to get a percentage. The economical feed factor was calculated by dividing the total kilogram of feed given with the total kilogram of slaughtered and sold fish. The share superior was calculated by dividing the number of fish classified as superior at slaughter by the total number of fish slaughtered.

We have obtained data from the two smolt facilities (Facility 1 and 2) producing “cold smolt” included in this study, health certificates, temperature records, feeding regimes, egg history and vaccine status.

There is little variation in the feeding and vaccination between the two hatchery facilities and other smolt producers. They have used standard feed from all four main feed producers in Norway, Skretting, Ewos, Biomar and Polarfeed, and commercial vaccines from the largest vaccine manufacturers in Norway, Elanco, Pharmaq and MSD as AlphaJectMicro 6, Aqua Vac PD7, Aquavac 6, Clynav. The eggs came from different sites operated by the three main egg producers MOWI, AquaGene and SalmoBreed. The smolt were bathed in probiotic bacteria (Stembiont^TM^) at the same time as vaccination for all smolt that went to production areas 3 and 4, but not for the smolt that went to production area 2.

### 2.2 Statistical analysis

We calculate cumulative probability: P(X≤x) whether the cold smolt groups performed than other groups in the same period and in the same area. The calculation was performed using the prerequisite we had a binominal distribution, with only two outcomes of each comparison that the cold smolt performed better or worse in relation to the groups they were compared with (Table 5).

This gives the cumulative probability that cold smolt performed better x or fewer times out of n comparisons:

$$P\left(X\geq x\right)=\sum_{k=x}^{n}\left(\begin{array}{c} n\\ k \end{array}\right)0.5^{k}{\left(1-0.5\right)}^{n-k}$$

*n* is the number of comparisons (e.g., cold smolt vs. other groups), and *x* is the number of times cold smolt performed better.

Since we use a binomial distribution with success probability of 0.5, the formula was simplified to:

$$P\left(X\geq x\right)=\sum_{k=x}^{n}\left(\begin{array}{c} n\\ k \end{array}\right){\left(0.5\right)}^{n}$$

## 3 Results

A total of 5.39 million “cold smolts” were sea-launched and included in this study (Table 1).

The “cold smolt” were compared with the other salmon smolt sea-launched at the same time and at the same locations. The “cold smolt” performed better at all sea-launches, locations and parameters (FCR, mortality, % superior) except % superior at site 6 sea-launched April 2020 (93 versus 94 %) and FCR at site 7 for salmon sea-launched August 2022 (Table 2).

Comparing of the “cold smolt” (1.52 million smolt) with the other groups (1.43 million smolt) from the three sites that all together had four cycles of groups from other smolt deliverers mainly 0-year old smolt (Table 3).

In Table 3 we summarize all 5.4 million of “cold smolt” that were followed and compared to the data of the average for the respective POs. The “cold smolt” perform better regarding mortality and FCR in all generations and locations compared to the average in these POs. The share of “superior” slaughter quality is also better during all year and all POs except fish harvested in 2019 and 2023 in PO2.

The “cold smolt” performed better in its range of mortality; from 2 to 74% better, FCR; from 4-22 % better and in the “superior” grouped product quality from 2 % worse to 49 % better.

## 4 Discussion

The results in this study support the previous observations/indications that salmon smolts produced slower, often in cold freshwater with natural light regimens, are performing better after sea-launch than smolts produced with protocols giving faster growth when they are in the freshwater grow-out stage (Frisk et al., 2020; Personal communication Ragnar Nystøyl, seminar Kristiansund, 17^th^ January 2025, Kontali).

The “cold smolt” performed better than the average conventionally produced smolt in all the analyzed parameters (FCRe, mortality, % superior) in all POs.

We also see that the “cold” salmon performed better than the other salmon at the same period at the same location (Table 3). Since the fish we are comparing with at the same location are also flow-through fish, the comparison is better than if it had been from RAS facilities, which have a completely different water chemistry. There are two groups and in relation to only one parameter in each group that the “other” salmon performed better than the “cold” smolt. One group had a weaker FCRe (179 000 salmon sea-launched in August 2022). The main explanation for this is probably that the harvest weight of the “cold” salmon was 2,0 kg heavier than the “other” smolt, and the FCR increase in large fish (Nordgarden et al., 2003). The other group of “cold” smolt released at the same site in August 2022 performed better than the “other” fish in all three parameters.

For the other group and its better parameter is the share of the “superior” quality at Location 6 sea-launched in April 2020, where the “cold smolt” had a share of 93 % and the other smolt a share of almost 94 %. Both groups were much better than the PO 4’s average (75.5 %).

The “cold smolt” was larger at sea launch at location seven and particularly at location nine. In location six the faster grown smolt, were larger at sea-launch.

The reason we wanted to look at production areas was that the southernmost areas had a considerably lower proportion of superior compared to the national average. This reduced level of superior classification is probably partly caused by a high level of sea lice in farmed salmon resulting in mechanical delousing (Sommerset et al., 2024).

The proportion of superior quality fish in Norway is historically lower in winter and spring than in summer and autumn (Sommerset et al., 2024). In our data set, the figures originated from primarily slaughtering in the autumn, late summer and early winter (around 2/3) while the last third was harvested in winter and spring. This will even out some of the differences that we saw on the “cold smolt” versus the average in the respective POs.

In location six, the cold produced fish performed below average regarding FCRe and mortality for the PO. However, the share of the “superior” product quality was good for the cold produced smolt. Information from that site revealed that both the “cold smolt” group and the external group got substantial challenges with the gills during the grow-out period. The “cold smolt” was slightly better than the conventional group in FCR and mortality, but slightly worse regarding share superior. The conventional group was larger at sea launch and was shorter time in sea and that was probably an advantage.

### 4.1 Light regimen, temperature and biorhythms

The light protocol (12/12, 10/14 – light/dark) employed in Facility 1 and 2 is more similar to the natural light in nature than the light protocol used by many smolt producers that employ more light during most of the production. The smolt producers often have continuous light until 2-3 months before smoltification (Striberny et al., 2021). The winter temperature of the water near 3 °C is also more similar to natural temperature than the temperature many fish farmers use (Stefanson et al., 2005).

The results in this study indicate that both light and temperature that are closer to the natural rivers should be used as test parameters in further analysis of production, welfare and health in salmon aquaculture. We cannot conclude from this study that only one of the two parameters or both are important based on our study design since we use both less light and lower temperature in parallel.

Some studies have been made on incubation temperature and growth performance in hatchery for Atlantic salmon. It has been concluded that growth in smolt production after hatching is increasing with increasing temperature up to 16°C (Dwyer and Piper, 1987; Stefansson et al., 2005).

We start to know something about the performance after sea-launch of smolt reared at different temperatures and light regimes. The studies indicate that colder water and varying water temperatures may be beneficial for the performance regarding growth (Flo et al. 2025; van Rijn et al., 2020; Lai 2024). In a trial where cardiac morphology was investigated, they found that smolt produced slowly (average 7.9 °C and 17,7 months) grew faster in the sea phase than smolt produced faster (12.5 °C and 11 months). The authors suggested that the cardiac morphology and the reduced cardiac capacity of the fast produced smolt could explain the reduced growth in the sea (Frisk et al., 2020). They found that the faster grown smolt in addition to slower growth rates at sea also had more pathological cardiac morphological alterations (Frisk et al., 2020).

By rapidly changing natural light and temperature variations, as in intensive smolt farming, one changes the biorhythms. It is not natural, in Norway, to have 12 degrees in the fresh water outside in winter, nor 24 hours of light.

### 4.2 Water and microbiome

All of the “cold-fish” in this survey were bred in flow-through freshwater sites without disinfection of the intake water, and all of the “cold fish” in PO 3 and 4 were given probiotic bacteria (Stembiont^TM^) in association with vaccination. About 1/3 of the 0-year old smolt released in the same location in the same period were also given the same probiotic bacteria. The probiotic bacteria may have contributed to improve the robustness of the smolt and could potentially be part of the reason for the better mortality rate, feed conversion rate and share of superior in the smolt we followed compared to the average of these regions. The *Aliivibrio* spp*.* used in the enhancement of the fish have earlier been shown to provide greater resistance to bacterial disease, less ulcers, lower mortality, lower FCR and increased resistance to salmon lice (Klakegg et al., 2020; Klakegg et al., 2020; Steen Dobloug, et al., 2023). The reason why probiotic bacteria are used in PO3 and 4 and not in PO2 could be that PO3 and 4 have larger problems and more need for probiotic enhancement than PO2. Nevertheless, also in PO2, the “cold fish” performed better than the average (Table 4).

**Table 4:** Performance of the “cold smolt” at sea compared to the average in the same year and production area in Norway. Green figures designate “cold smolt” performed better than the POs average, red figures designate “cold smolt” produced worse.

*PO1 and PO2 together. ** Numbers for whole Norway

**Table 5:** Cumulative probability: P(X≤x) that the cold smolt groups performed better than the average in respective POs each year^1^, where the cold smolt were cultivated together with another smolt at the same location and year compared to them^2^. We also compared the cumulative probability of the average of cold smolt within each PO each year compared to average in respective POs each year^3^. p was set at 0,5 (that the outcome if cold smolt would perform better than the group we compared with was random).

		Number of fish groups with cold smolt (n)	Numbers of fish groups of cold smolt better than the group we compare with (x)	Cumulative probability: P(X≥x)
^1^Compared to the average respective PO in the same year regarding:	FCR	18	17	0,00007
	mortality	18	16	0,00065
	share superior	18	16	0,00065
^2^Compared to the group with another smolt at the same site in the same year regarding:	FCR	6	5	0,10937^4^
	mortality	6	6	0,01562
	share superior	6	5	0,10937^4^
^3^The average of cold smolt within each PO each year compared to the average respective PO in the same year regarding:	FCR	10	10	0,00098
	mortality	10	10	0,00098
	share superior	10	8	0,05469^4^

We see that the groups with cold smolt performed significantly better (p>0,95) in all groups except three^4^.

We have also performed epigenetic analyses of five groups of “cold smolt” and compared the results with one group of 0-year-old smolt from an external smolt producer. The results from the epigenetic analysis revealed that several hundreds of genes were under different epigenetic control in the “cold smolt” compared with the smolt from external smolt producers (data not shown). Examples of genes and gene groups were HOX (developmental genes), stress genes: CIRBP (handles stress from light/darkness, hypoxia, temp), Calreticuline-like protein (protects other molecules from response to stress), immune genes, electrolyte and ion balance, cell surface genes, oxygen transport genes, energy and metabolism genes. All five groups of “cold smolt” had a significantly higher percentage of activated genes than the smolt from the external producer. Studies should be done to follow up on this, including experiments where we produce 0-year-smolt and 1.5-year-smolt from the same batch of eggs. The incubation regime in the egg phase should also be investigated with a focus on epigenetics.

“The cold” fish we investigated in this survey had a higher share of “superior” product quality category than both the average in the respective POs and the other fish at same time and same locations.

In recent years, the proportion of superior salmon from Norwegian slaughterhouses has had an evenly declining tendency from 91.0 % in 2017 to 83 % in 2023 (Riksrevisjonen, 2023; Sommerset, et al. 2024).

If we consider the time of slaughter, slightly more autumn slaughtered fish than the average for the respective POs, the performance is not as superior as the other parameters, but in total still substantial. The higher proportion of the “superior” product quality category we see in the cold smolt group overall compared to the average for respective regions is maybe due to a better adapted smolt. Immune genes were one of the groups of genes that was more active in the “cold smolt” group than the other smolt group (data not shown). The use of Stembiont^TM^ could also be part of the explanation since the use of probiotic *Aliivibrio* spp. reduces the incidence of ulcers and sea-lice infestation (Klakegg et al., 2020; Steen Dobloug, et al., 2023). Ulcers are one of the main reasons for downgrading the quality of harvested salmon (Sommerset et al., 2024).

Perhaps 10 or 12 hours of darkness in winter is still suboptimal and too much for the smolt. The egg phase of the life cycle of Atlantic salmon should also be studied regarding light, temperature and epigenetics. Compensation growth in salmon is a known mechanism where the fish adjust their growth rate to compensate for previously reduced growth potential in life, this could be part of the explanation for the potential high growth we also see indication of in the “cold smolt” (Hvas et al., 2022).

By removing the fish from the natural environment and creating a new environment, it is likely that this new environment is less suitable for the individual salmon than the environment the species has adapted to over time.

A freshwater phase where salmon are not produced as 0-year-old smolt, but have a more natural cycle is likely an important direction to a more sustainable salmon industry with a lower feed conversion factor, better survival and better animal welfare.

## 5 Conclusions

A total of 5.4 million of smolt sea-launched in PO 2,3 and 4 at the age of 15 months and older produced at cold water temperature in winter and with less light (“cold smolt”) than many smolt producers use, performed better regarding mortality, FCR and share of the “superior” product quality category than average in the respective POs, and also in comparison with other fish sea-launched at same time in the same locations.

## Ethic statement

### Journal of veterinary and animal sciences

Journal of Veterinary and Animal Sciences, is a journal whose articles are reviewed by peer reviewers in the field of veterinary and animal science. The Journal of Veterinary and Animal Sciences is dedicated to publishing scientific articles in the field of veterinary medicine and related matters. Journal of Veterinary and Animal Sciences is expected to become the premier registration and documentation of scientific work, in addition to being a veterinary discussion area.

The Journal of Veterinary and Animal Sciences should be able to ensure that all efforts made to publish scientific work have followed ethical rules of academics. Therefore, we consider it is very important to equate the views of the stakeholders in standard ethics in order to create a comfortable atmosphere and avoid the emergence of problems due to a conflict of interest in the future. This document is a policy statement taken by the Journal of Veterinary and Animal Sciences regarding the ethics of publication and malpractice to be used as a guide for stakeholders.

The Journal of Veterinary and Animal Sciences holds firmly to the standard publication ethics for all who involved in the publication of scientific papers, including authors, editors, reviewers, and publishers.

### Authors

Authors who do plagiarism are considered to behave disgraceful and less ethical. Authors are required to submit original works, not publishing them partially or fully to other journals, until the Journal of Veterinary and Animal Sciences gives an answer to the eligibility of the submitted article.

Each name listed in the article should be qualified as an author. Each author must have made important contributions to the article written, such as concepts, design, data collection, data analysis, interpretation of data, and decision making. For those who play an important role in the research but do not qualify as an author, his or her name can be listed in an acknowledgment.

The corresponding author is responsible and ensures that those who play an important role in research, their name have been listed as an author. Also, on behalf of the authors, the correspondence author should send a form (Form A) stating that they have fully accepted the last updated article and agreed to submit the article for publication.

### The author's responsibility

Authors are required to include original, accurate, and reliable research data. Authors are required to cite libraries that affect the article, be it an article in a print journal or online, or the results of personal interviews. The article of the authors should follow the review process by the reviewers that have been prepared by the Journal of Veterinary and Animal Sciences. The author is required to provide original and accurate data (without plagiarism and without fabrication) contained in the article and may provide additional data if necessary. Articles submitted to the Journal of Veterinary and Animal Sciences not sent to another journal, not being judged by a journal or even been accepted for publication. If a small piece of data has been published, the source should be cited or given a written thank you to the acknowledgment of the article. If the data is reprocessed from a source then proper citation and authorization are required. Research involving experimental animals should be conducted according to the rules of ethics and animal welfare. Any potential conflicts of interest shall be declared in respect of funding, personal research, or relationships with others or an organization that may influence the research. All funding sources that support the research or the sponsor of a study should be stated in acknowledgment. If the author finds and is aware of any error or error in his article, they must notify the chief editor, in order to be able to withdraw, clarify, and correct the article. If required, the authors should be willing to apologize for the mistakes they made. Requests to reduce, add or alter the author's arrangement to an accepted article should get the editor's approval and be done before the article is published.

### Animal ethics

Research involving experimental animals should be conducted in accordance with animal ethics and welfare. The animals should be well cared for and use as little as possible by minimizing the pain that may arise at the time of treatment. The use of experimental animals should be in accordance with local, national, and international rules. The authors must make a statement including the name of the ethical authority and the consent number, that the experiment has been carried out in compliance with animal ethics and welfare.

## CRediT authorship contribution statement

**Øystein Klakegg:** Writing – review & editing, Writing – original draft, Project administration, Methodology, Investigation, Formal analysis, Data curation, Conceptualization. **Henning Sørum:** Writing – original draft, Methodology, Investigation, Formal analysis, Conceptualization.

## Declaration of competing interest

The authors declare the following financial interests/personal relationships which may be considered as potential competing interests: Henning Sørum is one of the owners and founder of Previwo, the company that produces StembiontTM. If there are other authors, they declare that they have no known competing financial interests or personal relationships that could have appeared to influence the work reported in this paper.
